# Supplementary material for: Microgravity Impacts the Expression of Aging-Associated Candidate Gene Targets in the p53 Regulatory Network
Source: Int J Mol Sci. 2025 Nov 18;26(22):11140. doi: 10.3390/ijms262211140 (PMC12652855; doi:10.3390/ijms262211140)
Supplement: Supplementary file 1 [file ijms-26-11140-s001.zip › ijms-3825190-supplementary.pdf]

Table S1 RNA-seq data Quality Control summary

Sample name	Raw reads	Clean reads	Raw_data (G)	Clean_data (G)	Error_rate (%)	Q20 (%)	Q30 (%)	GC_content (%)
v1_bg	46796536	45047420	7G	6.8G	0.02	98.23	95.08	51.17
v1_7d	46724140	45443518	7G	6.8G	0.02	98.26	95.05	51.41
v1_14d	49098090	47808234	7.4G	7.2G	0.02	98.26	95.03	51.42
v1_21d	50853356	49543672	7.6G	7.4G	0.02	98.24	95.04	51.15
v1_28d	45631680	44183152	6.8G	6.6G	0.02	98.32	95.20	51.44
V2_bg	44888308	44070814	6.7G	6.6G	0.03	96.96	91.97	50.53
V2_7d	45780324	44950544	6.9G	6.7G	0.02	98.23	94.93	51.43
V2_14d	49696444	48885274	7.5G	7.3G	0.02	98.28	95.03	51.84
V2_21d	48890512	47973484	7.3G	7.2G	0.02	98.24	94.99	51.17
V2_28d	47399960	46252468	7.1G	6.9G	0.02	98.08	94.62	51.27
V3_bg	42573974	41761114	6.4G	6.3G	0.02	98.28	95.08	51.29
V3_7d	46350786	45183730	7G	6.8G	0.02	98.13	94.71	51.42
V3_14d	49702216	48891720	7.5G	7.3G	0.02	98.29	95.09	51.51
V3_21d	48054508	47100934	7.2G	7.1G	0.03	97.85	94.03	52.22
V3_28d	49432398	48650944	7.4G	7.3G	0.02	98.22	94.94	52.22
V4_bg	49481938	48596818	7.4G	7.3G	0.02	98.13	94.71	51.52
V4_7d	47134094	45654534	7.1G	6.8G	0.02	98.20	94.94	51.03
V4_14d	51958240	51008980	7.8G	7.7G	0.03	97.59	93.49	51.90
V4_21d	53697306	52809476	8.1G	7.9G	0.03	97.87	94.13	52.18
V4_28d	49796900	49047826	7.5G	7.4G	0.02	98.26	95.02	51.67
V5_bg	55237482	54768516	8.3G	8.2G	0.02	98.00	94.30	51.17
V5_7d	49361302	48825464	7.4G	7.3G	0.03	97.96	94.17	50.84
V5_14d	48631600	48096792	7.3G	7.2G	0.03	97.90	94.06	51.07
V5_21d	45971544	45540760	6.9G	6.8G	0.03	97.80	93.74	50.90
V5_28d	71499786	70731156	10.7G	10.6G	0.03	97.82	93.80	51.10

Table S1 (continued) RNA-seq data Quality Control summary

Sample name	Raw reads	Clean reads	Raw_data (G)	Clean_data (G)	Error_rate (%)	Q20 (%)	Q30 (%)	GC_content (%)
V6_bg	53495072	52919934	8G	7.9G	0.03	97.88	93.95	51.13
V6_7d	45084894	44596554	6.8G	6.7G	0.02	98.06	94.42	51.10
v6_14d	56037540	55554902	8.4G	8.3G	0.02	98.01	94.28	51.25
V6_21d	51215454	50711418	7.7G	7.6G	0.03	97.82	93.83	51.82
V6_28d	59565236	58929888	8.9G	8.8G	0.03	97.68	93.47	51.03
V7_bg	43780576	43160272	6.6G	6.5G	0.03	97.92	94.07	51.34
V7_7d	54201652	53636880	8.1G	8G	0.02	98.01	94.31	51.09
V7_14d	45768062	45288206	6.9G	6.8G	0.03	97.98	94.23	51.26
V7_21d	44871354	44441408	6.7G	6.7G	0.02	98.04	94.38	51.41
V7_28d	57929396	57350812	8.7G	8.6G	0.03	97.96	94.18	50.74
V8_bg	46252376	45748826	6.9G	6.9G	0.02	98.04	94.38	51.26
V8_7d	50537130	49962768	7.6G	7.5G	0.02	98.02	94.35	51.37
V8_14d	44930164	44472992	6.7G	6.7G	0.03	97.77	94.21	51.49
V8_21d	49597028	49134152	7.4G	7.4G	0.03	97.88	94.27	51.03
V8_28d	53691206	53138572	8.1G	8G	0.03	97.73	94.23	50.84
V9_bg	47871874	47407208	7.2G	7.1G	0.03	97.96	94.17	51.19
V9_7d	40271240	39819262	6G	6G	0.02	98.02	94.31	51.34
V9_14d	41146384	40766406	6.2G	6.1G	0.03	97.77	93.71	50.95
V9_21d	44522088	44035564	6.7G	6.6G	0.03	97.88	93.95	50.79
V9_28d	56435610	55731730	8.5G	8.4G	0.03	97.77	93.56	50.65
V10_bg	44473788	43926756	6.7G	6.6G	0.03	97.77	94.17	51.15
V10_7d	48830432	48333688	7.3G	7.3G	0.03	97.77	93.76	51.61
V10_14d	50709738	50073484	7.6G	7.5G	0.03	97.77	93.42	50.78
V10_21d	47444086	46812424	7.1G	7G	0.03	97.77	93.64	50.69
V10_28d	50713758	50213090	7.6G	7.5G	0.03	97.77	93.05	50.97

Table S2 **List of software used for RNA-seq data analysis**

Analysis	Software	Version	Parameters	Remarks
Mapping	Tophat	V2.0.12	mismatch = 2	mapping to a reference
Quantification	HTSeq	v0.6.1	-m union	
Differential Expression Analysis	DEGseq	v1.12.0	$\lvert log_2$Fold change$\rvert$ > 1; Padj < 0.005	DEGSeq used for samples without bioreplicates.
	EdgeR	3.0.8	Padj < 0.05	EdgeR used for specific conditions.
GO Enrichment	GOSeq, topGO, hmmscan	V2.12	Corrected P-Value<0.05	hmmscan
KEGG Enrichment	KOBAS	v3.0	Corrected P-Value<0.05	

Table S3 **Differential Gene Expression Analysis**

Type of Analysis	Software	Normalization	p-value estimation model	FDR estimation method
Differential Expression Analysis without bioreplicates	DEGseq	TMM	$\|\log_2$Fold change$\|$ > 1;<br>Padj < 0.005	BH
Comment	The readcount value of the ith gene in the jth sample is K_{ij}, then<br>Negative binomial distribution: K_{ij} NB(μ_{ij},$\sigma_{ij}2$)<br>Poisson distribution: K_{ij} P(μ_{ij})			

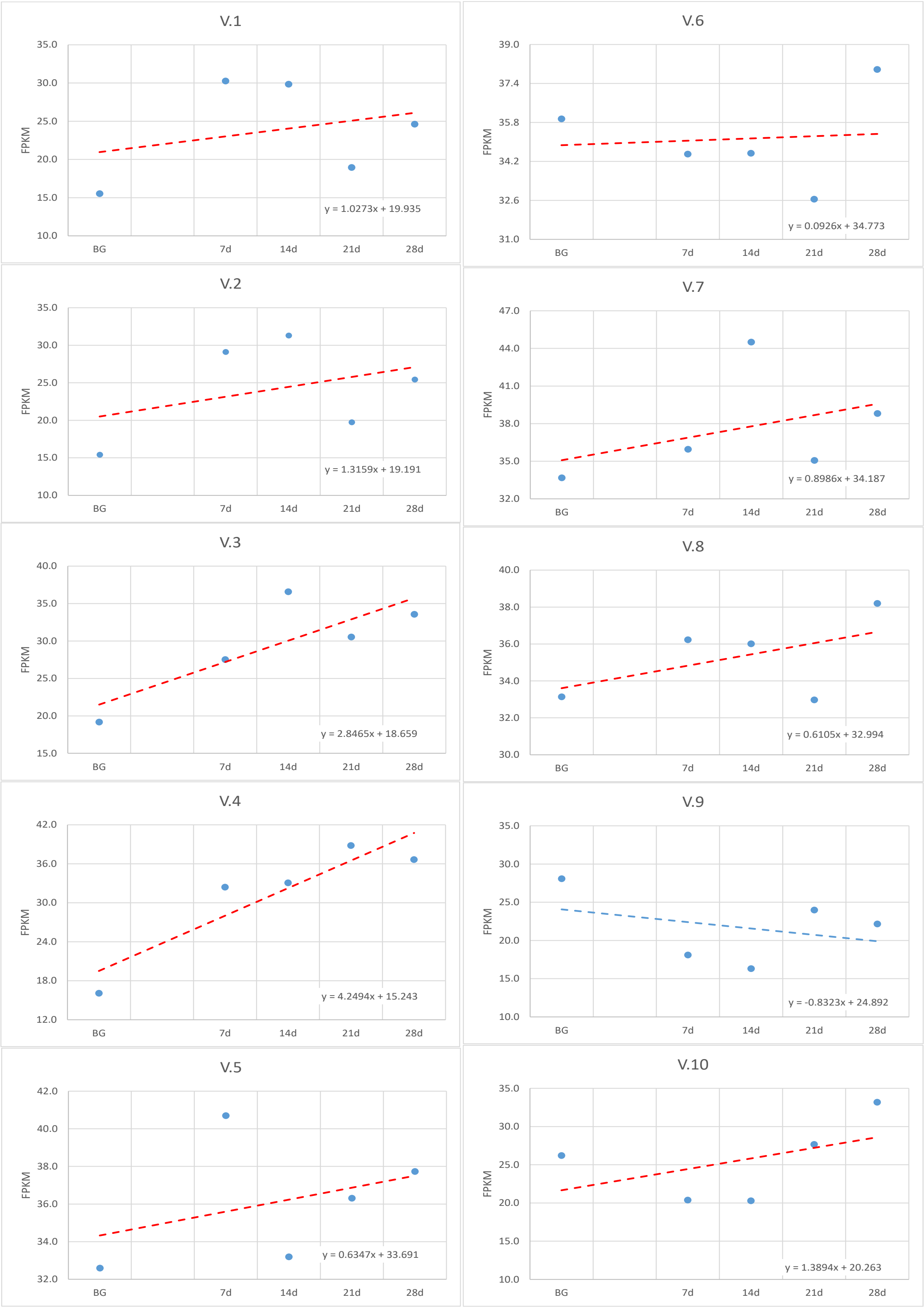

Supplementary Figure S1: Linear trend lines for p53 gene expression values (fragments per kilobase of transcript per million mapped fragments, FPKM)
Statistical analysis revealed a significant overall up-regulation of the TP53 tumor suppressor gene during the DI-SMG study (mean slope = 1.22 ± 0.88, 95% CI). This trend was consistent across the cohort, with elevated expression observed in T cell samples from 9 out of 10 volunteers.

Supplementary File S1 KEGG pathway enrichment analysis

Timepoint	#Term	Database	ID	Corrected P-Value	Hyperlink
7d_vs_bg	p53 signaling pathway	KEGG PATHWAY	hsa04115	4.19385E-04	https://www.kegg.jp/kegg-bin/show_pathway?hsa04115
14d_vs_bg	p53 signaling pathway	KEGG PATHWAY	hsa04115	5.50066E-04	https://www.kegg.jp/kegg-bin/show_pathway?hsa04115
21d_vs_bg	p53 signaling pathway	KEGG PATHWAY	hsa04115	8.48634E-05	https://www.kegg.jp/kegg-bin/show_pathway?hsa04115
28d_vs_bg	p53 signaling pathway	KEGG PATHWAY	hsa04115	1.04075E-03	https://www.kegg.jp/kegg-bin/show_pathway?hsa04115
